# *Longbie* capsules reduce bone loss in the subchondral bone of rats with comorbid osteoporosis and osteoarthritis by regulating metabolite alterations

**DOI:** 10.3389/fmed.2023.1256238

**Published:** 2023-10-17

**Authors:** Guihong Liang, Jinlong Zhao, Di Zhao, Yaoxing Dou, Hetao Huang, Weiyi Yang, Guanghui Zhou, Zhuoxu Gu, Jianke Pan, Jun Liu

**Affiliations:** ^1^The Second Clinical College of Guangzhou University of Chinese Medicine, Guangzhou, China; ^2^Guangdong Provincial Hospital of Chinese Medicine, Guangzhou, China; ^3^The Research Team on Bone and Joint Degeneration and Injury of Guangdong Provincial Academy of Chinese Medical Sciences, Guangzhou, China; ^4^The Fifth Clinical College of Guangzhou University of Chinese Medicine, Guangzhou, China; ^5^Guangdong Second Chinese Medicine Hospital (Guangdong Province Enginering Technology Research Institute of Traditional Chinese Medicine), Guangzhou, China

**Keywords:** geriatric medicine, bone aging, osteoarthritis, osteoporosis, Longbie capsule, plant-based natural products, metabonomics, bone metabolism

## Abstract

**Background and objective:**

With the development of global population aging, comorbidity (≥2 diseases) is a common health problem among elderly people. Osteoarthritis (OA) and osteoporosis (OP) are common in elderly individuals. There is a lack of drug therapy for OA and OP comorbidities. The purpose of this study was to explore the efficacy and mechanism of Longbie capsule (LBJN), which contains various plant herbs, in treating OA and OP comorbidities (OA + OP) in rats using metabolomics techniques.

**Methods:**

We created an OA + OP rat model through bilateral oophorectomy combined with meniscus instability surgery. Thirty SD rats were randomly divided into five groups (six in each group), namely, the sham group, OA group, OA + OP group, LBJN low-dose group (0.625 g/kg, OA + OP+LB-L group) and LBJN high-dose group (1.25 g/kg, OA + OP+LB-H group). After 8 weeks of intervention, we used micro-CT to detect bone microstructure status, ELISA to measure bone metabolism indicators, and UPLC–MS technology for metabolomics analysis. Finally, the screened differentially expressed metabolites were subjected to Kyoto Encyclopedia of Genes and Genomes (KEGG) pathway and functional enrichment analysis.

**Results:**

The micro-CT results showed that LBJN significantly improved the bone mineral density (BMD) and bone quality of subchondral bone in OA + OP rats, and LBJN regulated the expression of bone alkaline phosphatase (BALP), osteoprotegerin (OPG), and tartrate-resistant acid phosphatase (TRACP) in serum to maintain bone metabolism balance. Metabolomics analysis showed that the metabolic trajectory of OA + OP rats after intervention in the OA + OP+LB-H group showed significant changes, and 107 potential biomarkers could be identified. Among them, 50 metabolites were upregulated (such as *zeranol*) and 57 were downregulated (such as *vanillactic acid*). The KEGG functional enrichment results indicated that the differentially expressed metabolites are mainly involved in amino acid metabolism, lipid metabolism, and carbohydrate metabolism. The KEGG pathway enrichment results indicated that LBJN may exert therapeutic effects on OA + OP rats by regulating the cAMP signaling pathway, and the FoxO signaling pathway.

**Conclusion:**

LBJN can maintain bone metabolism balance by regulating serum lipid metabolism, amino acid metabolism, carbohydrate metabolism, and estrogen, thereby reducing bone loss in subchondral bone, which may be a potential mechanism through which LBJN treats OA + OP.

## 1. Introduction

Osteoporosis (OP) is a metabolic bone disease that results in a decrease in bone mass per unit volume due to multiple factors, leading to changes in bone microstructure and susceptibility to brittle fractures (1, 2). Osteoarthritis (OA) is a joint degenerative disease with degenerative articular cartilage, hypertrophic synovium and osteophytes as the main pathological changes (3, 4). The International Osteoporosis Foundation survey found that among the over 200 million people with OP worldwide, the majority are over 60 years old, and 9.6% of men and 18% of women over 60 years old worldwide may suffer from symptomatic OA. Age, sex, genetics, chronic inflammation, endocrine factors, and body mass index (BMI) are all considered common risk factors for OA and OP, and there may be complex correlations between the two (5, 6). A study reported that the proportion of the combined occurrence of OA and OP in postmenopausal women is as high as 56.5% (7), indicating a possible causal relationship between OA and OP. Al Saleh et al. (8) conducted a sampling survey of 3,985 adults and found that patients who had already developed knee osteoarthritis (KOA) were more likely to develop OP and that KOA accelerated the progression of OP. Dequeker et al. (9) found that OP can affect changes in overall bone mass, and abnormalities in the microstructure of subchondral bone tissue may lead to uneven stress on articular cartilage, which may lead to secondary cartilage damage and osteophyte proliferation, thereby promoting the occurrence and progression of OA. A case study found that a higher level of bone mineral density (BMD) can delay the progression of KOA (10). In this context, the merger of OA and OP (OA + OP) may be a serious challenge for future public health. However, there is no drug for targeted therapy of OA + OP. At present, the treatment of OA + OP mainly involves the combination of anti-inflammatory and analgesic drugs, anti-OP drugs, and cartilage-protective drugs (11–13), but this is clearly not the optimal treatment plan. Therefore, exploring drug therapies for OA + OP is an important task at present.

Traditional Chinese medicine has great potential advantages in treating OA + OP, and it has more possibilities for drug conversion. Our previous evidence-based research has confirmed that kidney-tonifying and blood-activating traditional Chinese medicine has a definite therapeutic effect on treating KOA (14). In addition, the kidney-tonifying and blood-activating method is also recommended in the treatment of OP (15). The kidney-tonifying and blood-activating pharmacological effects of traditional Chinese medicine on bones are multifaceted (15–17). These medicines can directly increase the activity of osteoblasts to promote osteoblast regeneration and correct the dysfunction of the immune system to protect gonadal tissue while maintaining sex hormone levels and bone metabolic balance. Kidney-tonifying and blood-activating herbs can also affect the bone remodeling cycle and bone resorption cycle (18, 19). Under the kidney-tonifying and blood-activating theory in traditional Chinese medicine, Guangdong Provincial Hospital of Chinese Medicine has developed the Longbie capsule (LBJN), which contains various plant herbs and has achieved good effects in treating KOA. The main components of LBJN are Xianmao (*Curculigo orchioides Gaertn.*), Bajitian (*Morinda officinalis How.*), Wugong (*Scolopendra subspinipes*), Qishe (Agkistrodon), Quanxie (Scorpio), Tusizi (*Cuscuta chinensis*), Dansen (*Salvia miltiorrhiza Bunge*), Tubiechong (*D.dispar Chanisso et Eysenhard*), Chuanwu (*Aconitum carmichaelii*), and Huangqi (*Astragalus membranaceus (Fisch.) Bunge*). However, the efficacy and mechanism of LBJN in treating OA + OP are still unclear.

Metabonomics is a systematic study of the changing levels of metabolites and is widely used to evaluate the efficacy and potential pharmacological mechanisms of natural products (20). The composition, concentration and structure of metabolites can be obtained through metabonomic detection, and more information can be provided for systems biology research on the basis of further supplementing gene, transcriptional and protein information. The comprehensive, systematic and dynamic characteristics of metabonomics are very similar to the holistic theory of traditional Chinese medicine, which provides a new dimension for the study of the holistic effect of traditional Chinese medicine (21). The purpose of this study was to explore the efficacy and mechanism of LBJN in the treatment of OA + OP using metabolomics methods.

## 2. Materials and methods

### 2.1. Animals and ethical approval

A total of 30 SPF-grade female SD rats aged 1 month and weighing 100–120 g were included in this experiment. These rats were purchased from the Guangdong Medical Experimental Animal Center (certificate number: 44007200103590). These SD rats were raised in an SPF environment at the Experimental Animal Center of Guangdong University of Chinese Medicine. The experimental plan was approved by the Animal Ethics Committee of Guangdong Provincial Hospital of Chinese Medicine (ethical approval number: 2021028). The procedures for this experiment were performed in accordance with the regulations on the administration of experimental animals approved by the State Council of the People’s Republic of China.

### 2.2. Animal model construction and intervention measures

All 30 SD rats were divided into five groups, with six rats in each group: the sham group, OA group, OA + OP group, LBJN low-dose group (0.625 g/kg, OA + OP+LB-L group), and LBJN high-dose group (1.25 g/kg, OA + OP+LB-H group). LBJN (batch number: YueYaoZhiZi Z20071030, 0.5 g/capsule) was produced and provided by the Pharmacy Center of Guangdong Provincial Hospital of Chinese Medicine. In addition, the LBJN dose in this study follows the human mouse dose conversion formula, and low- and high-dose groups were formed.

We used isoflurane to anaesthetize SD rats. Except for the sham group, all four groups of rats underwent surgical resection of the medial meniscus and tibial collateral ligament of the knee joint to construct an OA model of meniscus instability. In the sham group, the knee joint capsule was separated and then sutured. Except for the sham group and the OA group, all rats in the other groups underwent surgical resection of both ovaries to construct an OP model. In the sham group and OA group, both ovaries were separated and resutured after removing some adipose tissue around both ovaries. Starting 4 weeks after surgery, medication was administered by gavage. LBJN was dissolved in pure water and made into a mixture of traditional Chinese medicines. The medication program for the OA + OP+LB-L and OA + OP+LB-H groups was administration once a day by gavage and continuous gavage for 8 weeks. The other three groups were given the same dose of pure water by gavage. After 8 weeks of gavage, the rats were euthanized under anesthesia, and the serum and knee joints of each group of rats were collected for further analysis.

### 2.3. Chemical composition analysis of LBJN

We used high-performance liquid chromatography quadrupole/electrostatic field orbital trap high-resolution mass spectrometry (HPLC-Q-Orbitrap-MS) technology to identify the chemical components in LBJN aqueous solution. The main instruments used for component identification included a Q Exactive high-resolution mass spectrometer (Thermo Fisher Scientific), Ultimate 3000RS ultra-high-performance liquid chromatograph (Thermo Fisher Scientific), and Welch AQ-C18 chromatography columns (2.1 mm × 150 mm, 1.8 μm).

Sample processing was performed as follows: 200 μL of LBJN aqueous solution was added to 1,000 μL of 80% methanol and vortexed for 10 min. The samples were centrifuged for 10 min at a temperature of 4°C with a centrifugal force of 20,000 × g, and the supernatant was filtered for analysis.

The mass spectrum (MS) conditions were as follows: the ion source was an electric spray ionization source (ESI); positive and negative ion switching scanning; full mass/dd-MS2 detection method for detection; resolution of 70,000 (full mass), 17,500 (dd-MS2); scanning range of 100.0–15,000.0 m/z; electric spray voltage of 3.2 kV; capillary temperature of 300°C; collision gas of high-purity argon gas (purity ≥99.999%); sheath gas of nitrogen (purity ≥99.999%), 40 Arb; auxiliary gas of nitrogen (purity ≥99.999%), 15 Arb, 350°C; and data collection time of 30.0 min.

The chromatographic conditions were as follows: a Welch AQ-C18 column (2.1 mm × 150 mm, 1.8 μm) was used; the flow rate was 0.30 mL/min; the aqueous phase was 0.1% formic acid aqueous solution; the organic phase was methanol; the temperature of the column temperature box was 35°C; the temperature of the automatic sampler was 10.0°C; and the injection volume of the automatic sampler was 5.00 μL.

All data collected through high-resolution liquid quality methods was preliminarily organized using Compound Discoverer 3.3 software (Thermo Fisher Scientific) and compared and analyzed in the mzCloud database. Finally, we analyzed the various spectral peaks and further inferred the compound structure based on the ion fragments and retention information provided by references and databases.

### 2.4. Micro-CT analysis

After fixing the knee joint sample with 4% paraformaldehyde, we used a micro-CT scanner (ZKKS-MCT-Sharp, Guangzhou, China) to scan and analyze the imaging morphology of the femur. During scanning, the knee joint was fixed on the fixator along the long axis. The scanning voltage was set to 70 kV, and the current was 100 μA. The power was 7 W, 4 frames were stacked, the angle gain was 0.72 degrees, scanning was completed by rotating one cycle, and the scanning layer thickness was 15 μM. ZKKS-MicroCT 4.1 software was used to analyze the bone morphology-related parameters of the tibial subchondral bone, including the BMD, bone volume fraction (BV/TV), trabecular number (Tb.N), bone surface area to bone volume ratio (BS/BV), trabecular thickness (Tb.Th), and trabecular separation (Tb.Sp).

### 2.5. Analysis of bone metabolic factors

All blood samples from SD rats were collected and centrifuged at 1500 rpm for 15 min. These serum samples were stored in an environment of −80°C. We used the enzyme-linked immunosorbent assay (ELISA) method to detect the expression levels of serum bone alkaline phosphatase (BALP), osteoprotegerin (OPG), bone gla protein (BGP) and tartrate-resistant acid phosphatase (TRACP). The measurement process was carried out according to the instructions of the ELISA kit (Jiangsu Meimian Industrial Co., Ltd., Jiangsu, China).

### 2.6. Metabolite analysis and data processing

Ultra-high-performance liquid chromatography–mass spectrometry (UPLC–MS) technology was applied for the analysis of serum metabolites. The instrument platform for LC–MS analysis was the UHPLC-Q Exactive system manufactured by Thermo Fisher Scientific.

For the MS, the optimal conditions were set as follows: heater temperature, 400°C; capillary temperature, 320°C; sheath gas flow rate, 40 arb; Aux gas flow rate, 10 arb; ion-spray voltage floating (ISVF), −2,800 V in negative mode and 3,500 V in positive mode; and normalized collision energy, 20–40-60 V rolling for MS/MS. Data acquisition was performed in data-dependent acquisition (DDA) mode. The detection was carried out over a mass range of 70–1,050 m/z.

The chromatographic conditions were as follows: 2 μL of sample was separated by an HSS T3 column (100 mm × 2.1 mm i.d., 1.8 μm) and then subjected to mass spectrometry detection. The mobile phases consisted of 0.1% formic acid in water:acetonitrile (95:5, v/v) (solvent A) and 0.1% formic acid in acetonitrile:isopropanol:water (47.5:47.5:5, v/v) (solvent B). The sample injection volume was 2 μL, and the flow rate was set to 0.4 mL/min. The column temperature was maintained at 40°C. During the period of analysis, all samples were stored at 4°C.

All collected data were first preprocessed, which included missing value recoding and normalization of original data. The processed data were imported into SIMCA-P 11.5 software for principal component analysis (PCA), supervised partial least squares discriminant analysis (PLS-DA), and orthogonal partial least squares discriminant analysis (OPLS-DA), and ions that met the criteria of variable importance in projection (VIP) values ≥1 and *p* < 0.05 were screened as differentially expressed metabolites. The score plot results were used to obtain sample classification information. A loading plot was used to screen for differential metabolic molecules. We classified the differentially expressed metabolites obtained through the internationally recognized Human Metabolome Database (HMDB).^1^ SciPy (Python, version 1.0.0) software was applied to draw differentially expressed metabolite heatmaps and to screen for differentially expressed metabolites based on *p* < 0.05. Finally, we imported the obtained differentially expressed metabolites into the Kyoto Encyclopedia of Genes and Genomes (KEGG) Compound (Release 2017-05-01) and KEGG Pathway (Release 2017-05-01) for metabolic pathway analysis.

### 2.7. Data analysis

SPSS 17.0 software and GraphPad Prism 5.0 were applied for data analysis and result visualization. The measurement data are expressed as the mean ± standard deviation. The *t*-test was used for intergroup comparisons, and *p* < 0.05 was considered statistically significant.

## 3. Results

### 3.1. Chemical composition of LBJN

**Figure 1:**
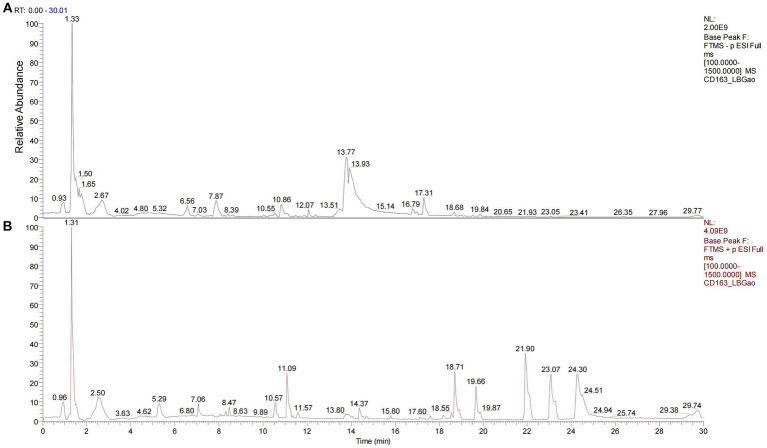
Chromatogram of LBJN based on Q-Orbitrap high-resolution liquid mass spectrometry. **(A)** Negative ion mode. **(B)** Positive ion mode.

We used HPLC-Q-Orbitrap-MS technology to collect MS data for LBJN. The fundamental peak chromatogram of LBJN in positive and negative ion modes is shown in Figure 1. The data collected based on HPLC-Q-Orbitrap-MS technology were processed using Compound Discoverer 3.3 software (Thermo Fisher Scientific) and the mzCloud database, and a total of 515 compounds were matched (Supplementary material 1). The results showed that potential compounds within LBJN that could play pharmacological roles include tanshinone IIA, oleanolic acid, albiflorin, rubiadin, trigonelline, cryptotanshinone, berberine, catechin, and neochlorogenic acid. Table 1 shows the common compounds through which LBJN exerts pharmacological effects.

**Table 1:** Partial results of compound identification in LBJN.

Name	Formula	Annot. Source: Predicted Compositions	Annot. Source: mzCloud Search	Annot. Source: MassList Search	Annot. DeltaMass [Da]	Calc. MW	RT [min]	mzCloud Results	mzCloud Best Match
Oleanolic acid	C30 H48 O3	Not the top hit	Full match	Partial match	−0.0005	456.3599	22.112	3	99.9
Tanshinone IIA	C19 H18 O3	Full match	Full match	Partial match	−0.0005	294.1251	19.671	7	99.7
Rubiadin	C15 H10 O4	Full match	Full match	Partial match	−0.00006	254.0578	18.509	4	99.6
Trigonelline	C7 H7 N O2	Full match	Full match	Partial match	−0.00008	137.0476	1.45	9	99.4
Cryptotanshinone	C19 H20 O3	Full match	Full match	Partial match	−0.00074	296.1405	18.719	3	99.3
Berberine	C20 H17 N O4	Not the top hit	Full match	Partial match	−0.00048	335.1153	11.685	9	97.9
Neochlorogenic acid	C16 H18 O9	Not the top hit	Full match	Partial match	−0.00013	354.095	8.596	2	97.2

### 3.2. The effect of LBJN on the subchondral bone structure

**Figure 2:**
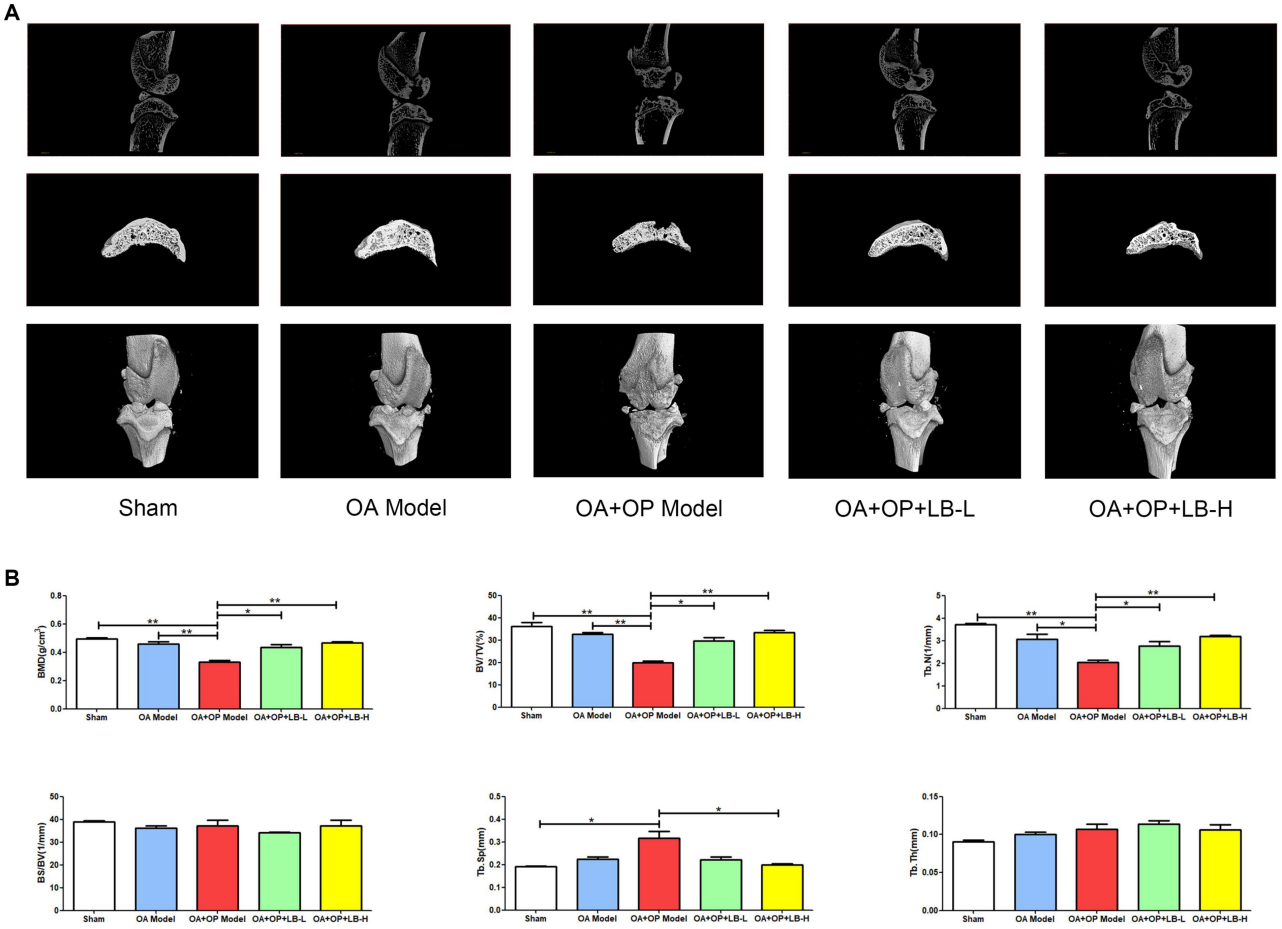
Therapeutic effects of LBJN on the bone histomorphometry of subchondral bone in the OA and OP model. **(A)** Micro-CT images of the bone structure in the sham, OA model, OA + OP model, OA + OP+LB-L, and OA + OP+LB-H groups (*n* = 4). **(B)** Morphometric analysis of BMD, BV/TV, Tb.N, BS/BV, Tb.Th, and Tb.Sp in subchondral bone of the knee joint. *p < 0.05, **p < 0.01.

The micro-CT images of the bone structure can be found in Figure 2A. The micro-CT results showed that at 12 weeks after surgery, the BV/TV, BMD, and Tb.N in the OA + OP group were significantly lower than those in the sham and OA groups (Figure 2B), while the Tb.Sp was significantly higher than that in the sham group, indicating significant bone loss in the subchondral bone of the knee joint in OA + OP rats. The BV/TV, BMD, and Tb.N of the OA + OP+LB-L group and the OA + OP+LB-H group were higher than those of the OA and OA + OP groups; the Tb.Sp values of the OA + OP+LB-L group and the OA + OP+LB-H group were lower than those of the OA and OA + OP groups. The above results indicate that the decreases in BMD and bone mass of the subchondral bone of the tibia in the disease state of OA combined with OP are significantly greater than those in OA alone. LBJN can improve the BMD and bone mass of subchondral bone (Figure 2B), indicating that LBJN may have potential drug therapeutic value for OA + OP.

### 3.3. The effect of LBJN on bone metabolic factors

**Figure 3:**
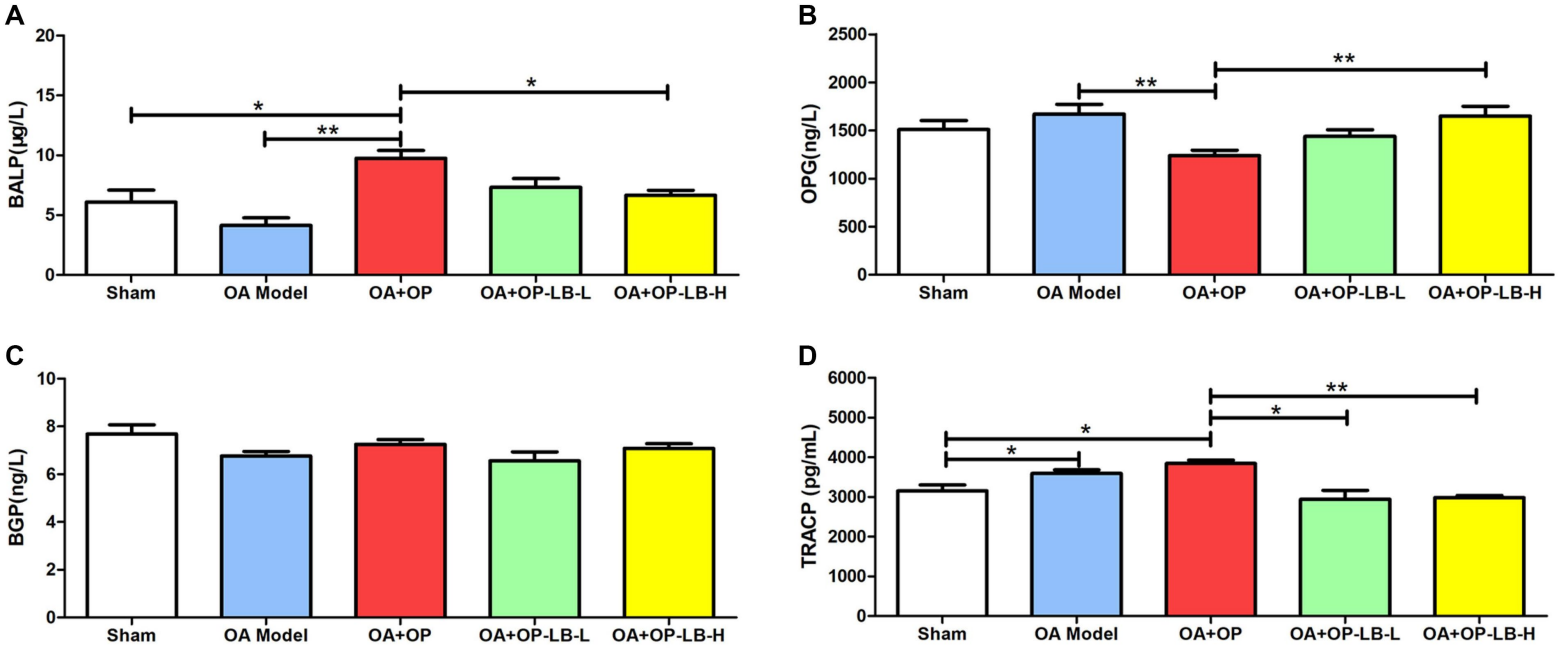
Therapeutic effects of LBJN on the serum concentrations of **(A)** BALP, **(B)** OPG, **(C)** BGP, and **(D)** TRACP in the OA and OP model (*n* = 6). *p < 0.05, **p < 0.01.

We used ELISA to detect serum bone metabolism indicators in the rats in each group. The results showed that the BALP expression level in the OA + OP group was significantly higher than that in the sham and OA groups, while that in the OA + OP+LB-H group was significantly lower than that in the OA + OP group (Figure 3). During bone formation, BALP is released by osteoblasts and plays a role in promoting bone formation and bone matrix mineralization (22). When bone volume decreases, meaning Tb.N decreases, the body experiences compensatory bone formation and an increase in serum BALP levels (23). The increase in serum BALP levels reflects an increase in bone formation and also implies higher bone turnover, depletion of the body’s ability to regenerate osteoblasts, accelerated apoptosis of osteoblasts, and exacerbation of the condition, leading to a continuous decrease in bone volume (24). Therefore, we can observe that the expression level of BALP in the OA + OP group is higher than that in the sham group. The expression level of OPG in the OA + OP group was significantly lower than that in the OA group, while that in the OA + OP+LB-H group was significantly higher than that in the OA + OP group (Figure 3). There was no statistically significant difference in the BGP expression levels among the groups (*p* > 0.05). The expression levels of TRACP in the OA and OA + OP groups were significantly higher than those in the sham group, while those in the OA + OP+LB-L and OA + OP+LB-H groups were significantly lower than those in the OA + OP group (Figure 3). The above results indicate that LBJN can affect bone metabolism in OA + OP rats by regulating the expression of serum BALP, OPG, and TRACP.

### 3.4. The effect of LBJN on serum metabolites

**Figure 4:**
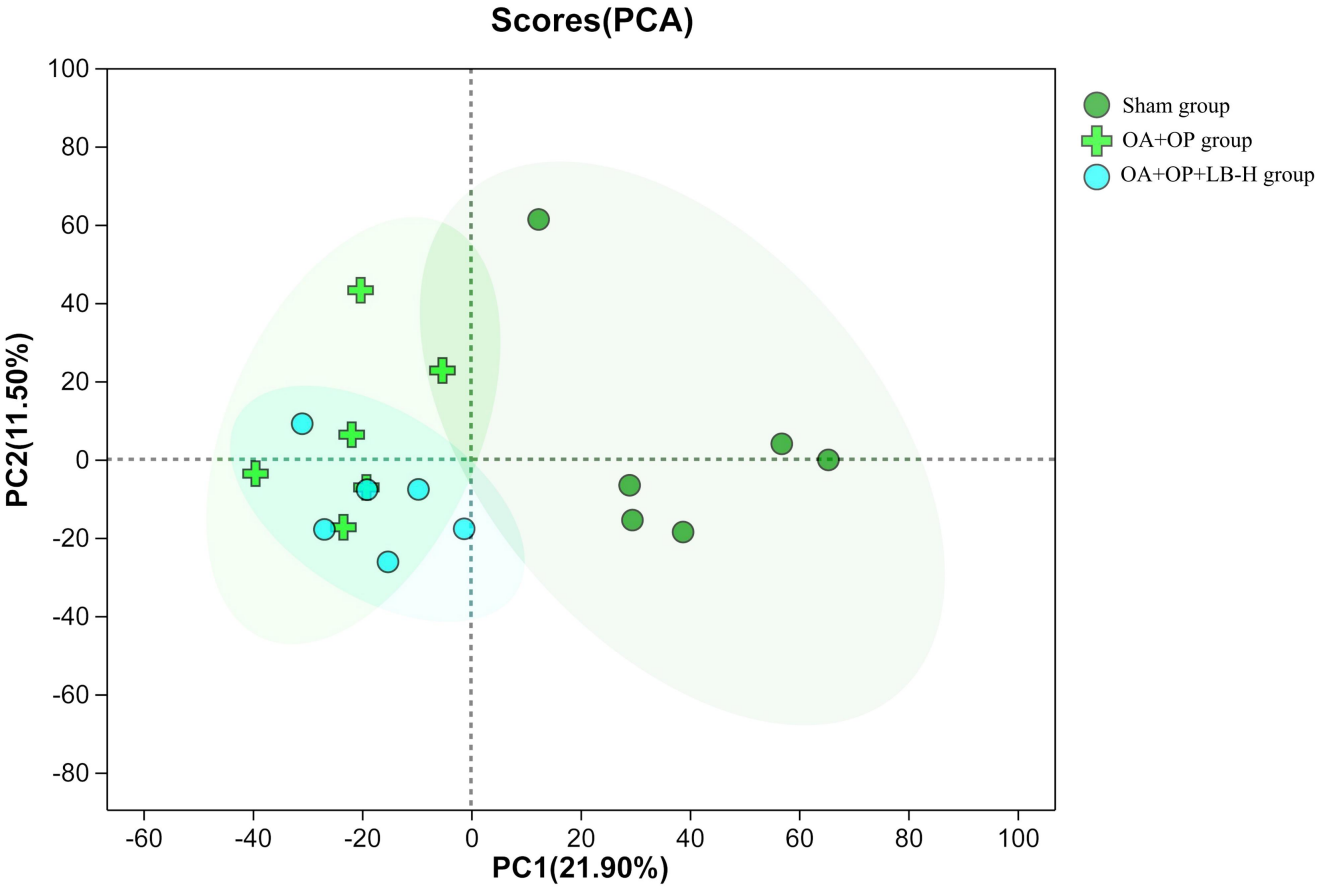
Principal component analysis score chart for the OA + OP, OA + OP+LB-H, and sham groups.

**Figure 5:**
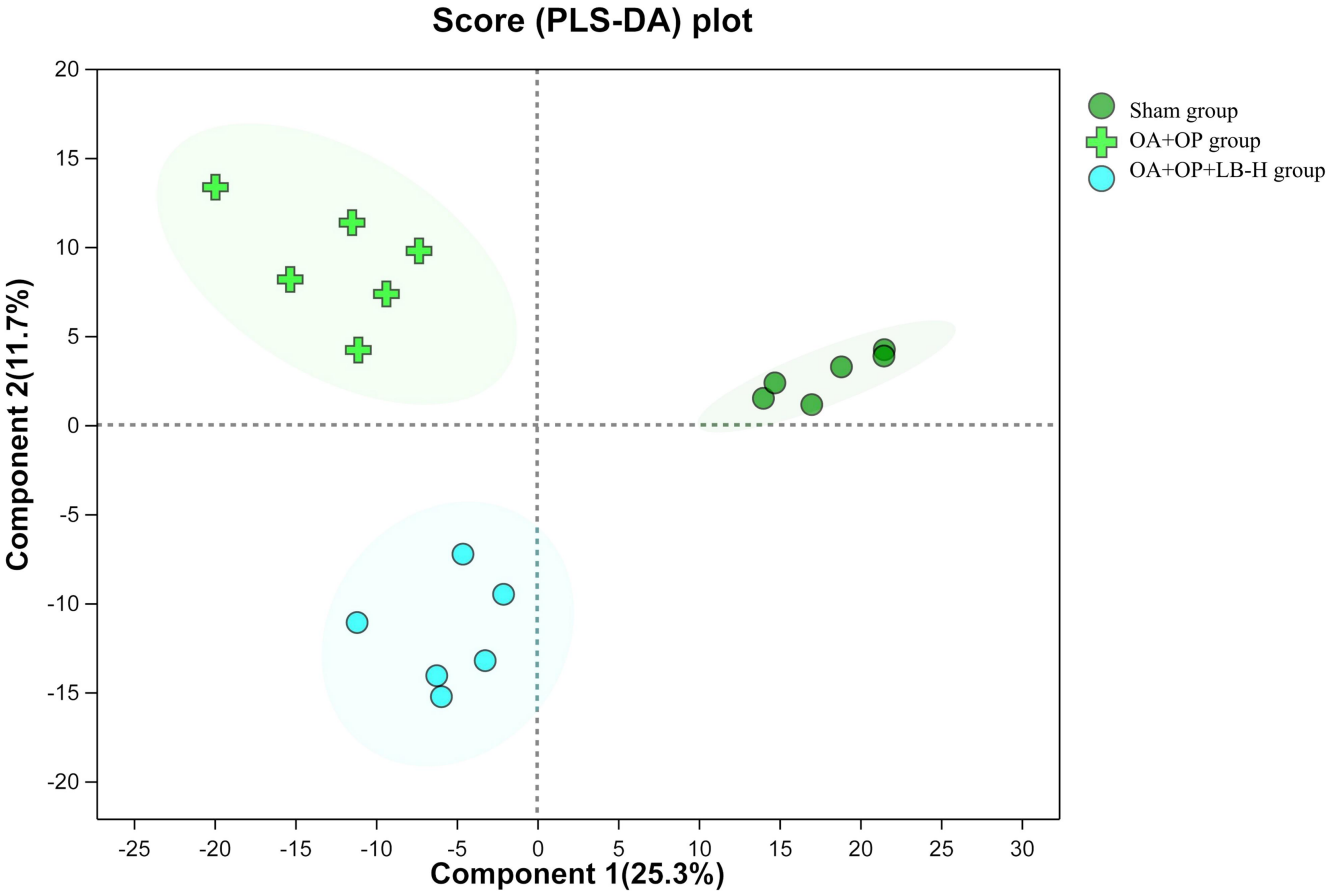
Partial least squares discriminant analysis scores for the OA + OP, OA + OP+LB-H, and sham groups.

We used a nontargeted metabolomics strategy to investigate the effect of high-dose LBJN (1.25 g/kg) on endogenous serum metabolites in OA + OP rats. Using multivariate statistical analysis methods, PCA and OPLS-DA were performed on the data from the OA + OP and OA + OP+LB-H groups, and corresponding identification models were established. Based on this information, differentially expressed metabolites were identified. The PCA results showed that there were no significant differences in the metabolic profiles among the OA + OP, OA + OP+LB-H, and control groups (Figure 4). The prediction rates of PC1 and PC2 did not reach 50%, so the difference between two groups could not be identified. The PLS-DA results showed that the OA + OP, OA + OP+LB-H, and control groups were completely separated (Figure 5). There were differences in the distribution of metabolites in the body of OA + OP rats compared to the other two groups (OA + OP group and OA + OP+LB-H group, OA + OP group and control group).

**Figure 6:**
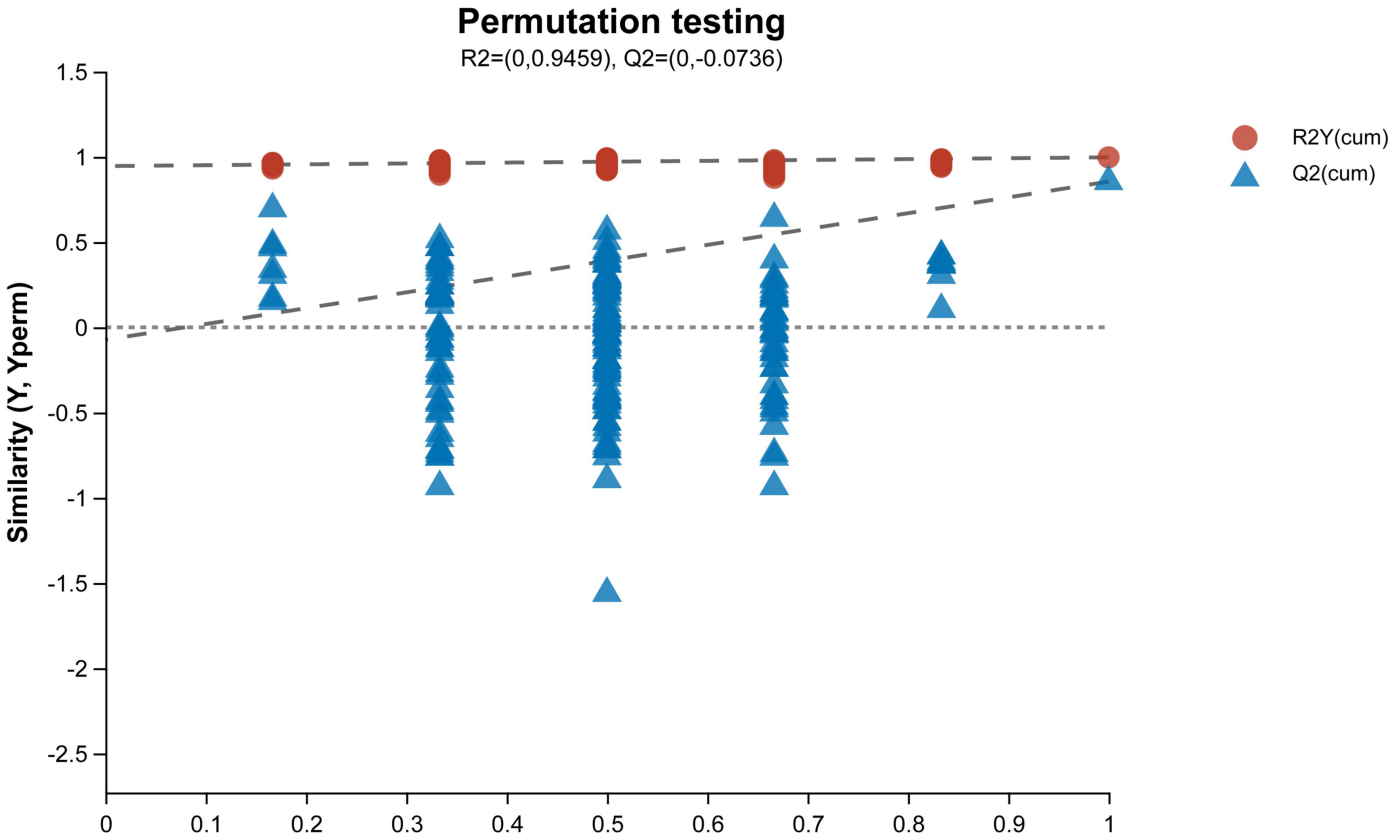
Permutation test diagram of the OPLS-DA model. The abscissa represents the replacement retention of the permutation test (the proportion is consistent with the order of the Y variable of the original model, and the point with a replacement retention of 1 is the R2 and Q2 value of the original model); the ordinate represents the R2 (red dot) and Q2 (blue triangle) values obtained by the permutation test, and the two dotted lines represent the regression lines of R2 and Q2, respectively.

The results of the permutation test evaluation of the OPLS-DA model are shown in Figure 6. The Q2 value of the permutation test random model is less than the Q2 value of the original model, and the intercept between the regression line of Q2 and the vertical axis is less than zero, which indicates that the original model has good robustness and no overfitting phenomenon. Therefore, based on a reliable evaluation model, we found significant differences in metabolites between the OA + OP group and the control group, and there were also significant changes in metabolites after LBJN intervention.

**Figure 7:**
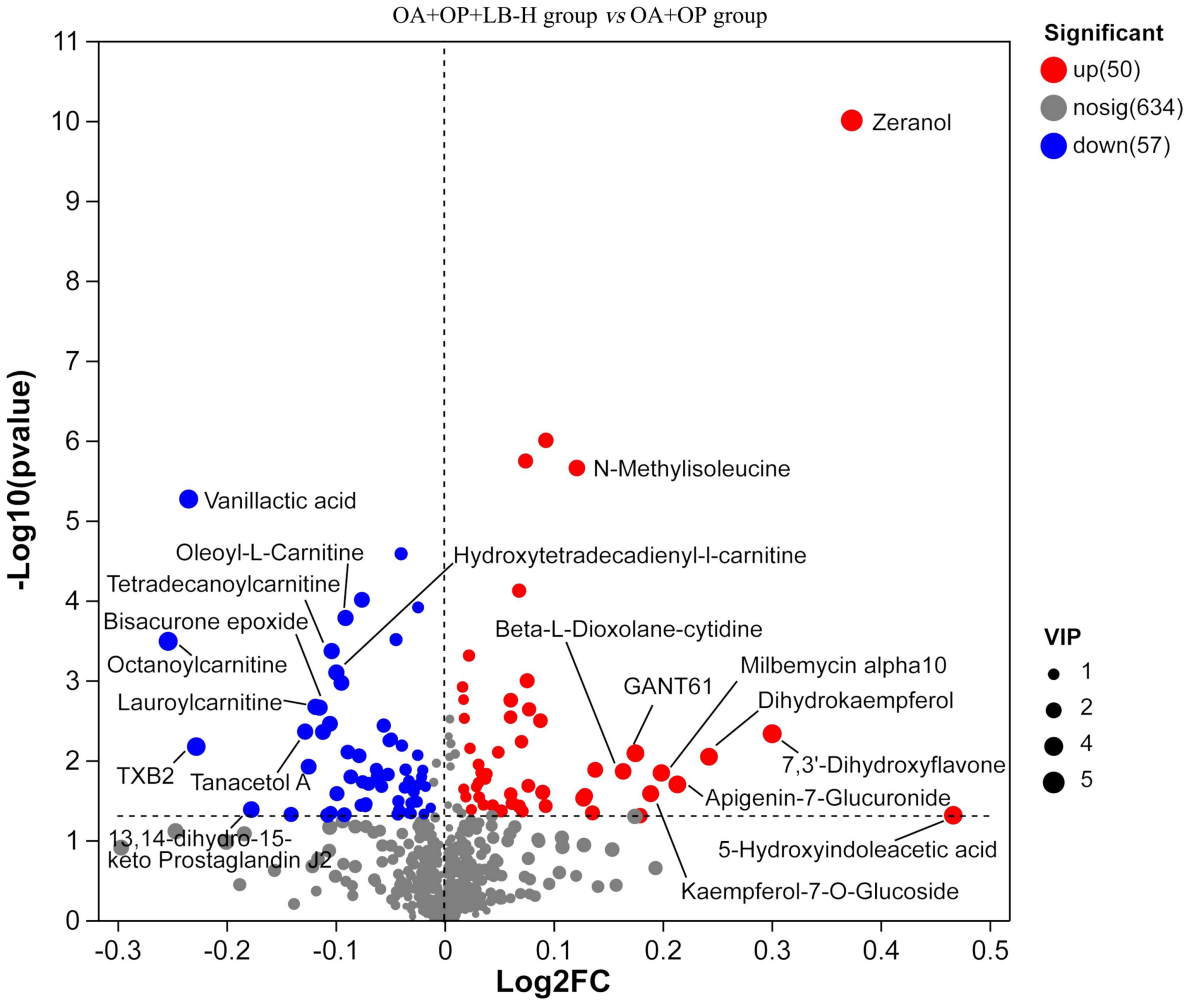
Volcanic maps of the metabolic differences between the OA + OP and OA + OP+LB-H groups. The size of the circle represents the variable importance in projection (VIP) value, the scatter color represents the final screening result, significantly upregulated metabolites are represented in red, significantly downregulated metabolites are represented in blue, and non-significantly different metabolites are gray.

**Figure 8:**
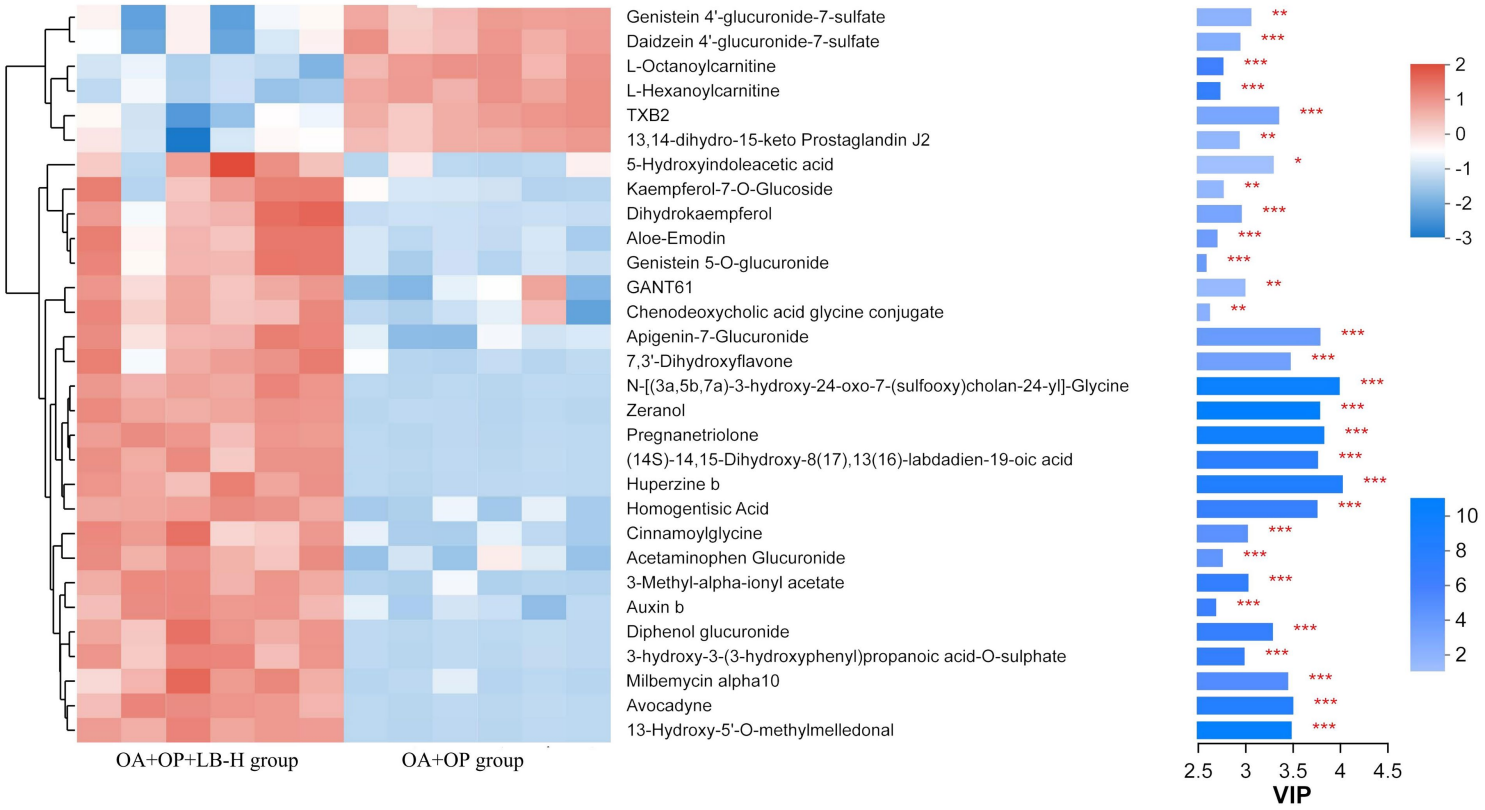
VIP value analysis of metabolites in the OA + OP and OA + OP+LB-H groups. *p < 0.05, **p < 0.01, ***p < 0.001.

In positive ion mode, a total of 107 differentially expressed metabolites were screened (Figure 7), and detailed information on these 107 metabolites is shown in Supplementary material 2. Compared with the OA + OP group, the OA + OP+LB-H group had a total of 50 differentially expressed metabolites that were upregulated and 57 metabolite levels that were downregulated. According to the magnitude of the VIP values, Table 2 shows a total of 10 metabolites with the most significant upregulation and downregulation. Through differentially expressed metabolite volcano maps, it can be found that points farther away from the center are more likely to become potential biological targets (Figure 7). The VIP value analysis results showed that compared with the OA + OP group (Figure 8), the OA + OP+LB-H group showed significant upregulation of metabolites such as eranol, 7,3′-dihydroxyflavone, and 5-hydroxyindoleacetic acid (*p* < 0.001), while vanillactic acid, octanoylcarnitine, and TXB2 were significantly downregulated (*p* < 0.001).

**Table 2:** Main differential metabolic product information.

Metabolite	Regulate	VIP value
Zeranol	Up	5.4731
7,3′-Dihydroxyflavone	Up	4.0761
5-Hydroxyindoleacetic acid	Up	3.8894
GANT61	Up	3.6068
Apigenin-7-Glucuronide	Up	3.5811
Dihydrokaempferol	Up	3.5093
Milbemycin alpha10	Up	3.4012
Kaempferol-7-O-Glucoside	Up	3.2909
N-Methylisoleucine	Up	3.0862
Beta-L-Dioxolane-cytidine	Up	3.0578
Vanillactic acid	Down	4.071
Octanoylcarnitine	Down	4.0155
TXB2	Down	3.8165
13,14-dihydro-15-keto Prostaglandin J2	Down	3.1308
Tetradecanoylcarnitine	Down	2.9628
Tanacetol A	Down	2.9463
Lauroylcarnitine	Down	2.9399
Oleoyl-L-Carnitine	Down	2.9321
Bisacurone epoxide	Down	2.8844
Hydroxytetradecadienyl-l-carnitine	Down	2.8801

**Figure 9:**
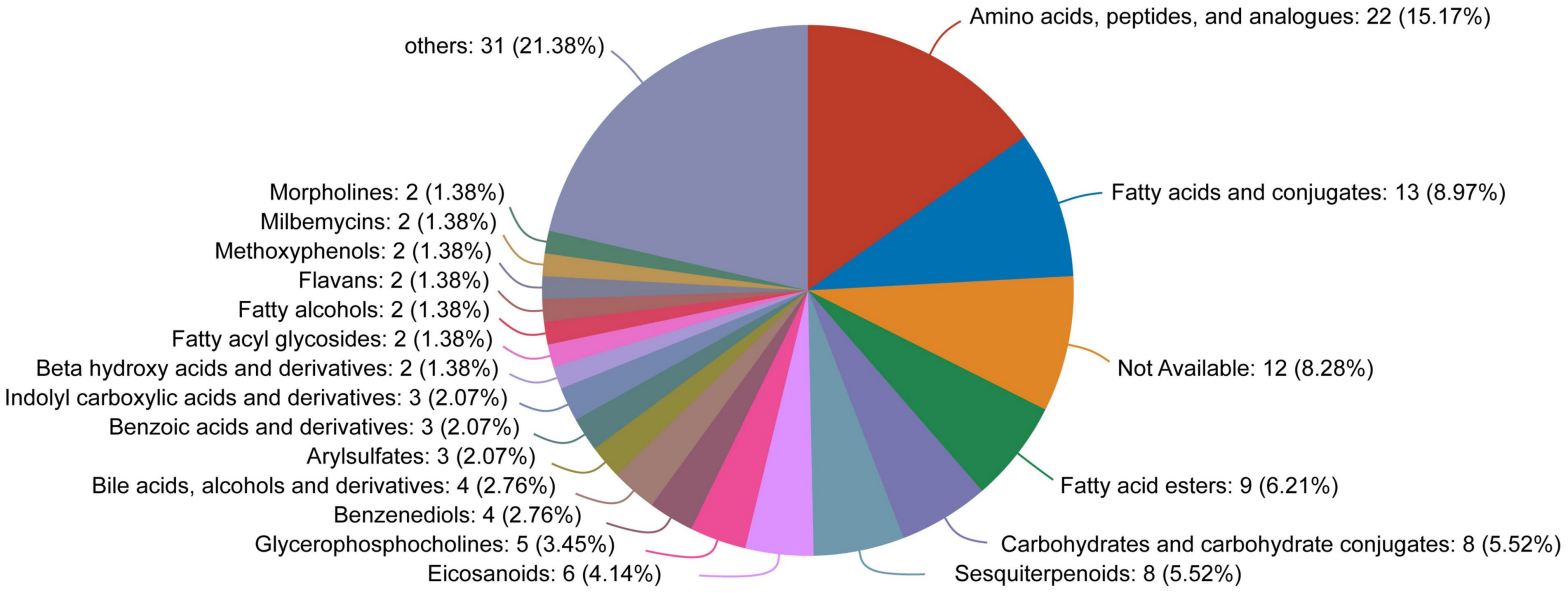
HMDB compound classification diagram for the differentially expressed metabolites between the OA + OP and OA + OP+LB-H groups.

By comparing to the HMDB, we found that the differentially expressed metabolites were mainly classified as amino acids, peptides, and analogs (15.17%); fatty acids and conjugates (8.97%); and fatty acid esters (6.21%) (Figure 9).

### 3.5. KEGG analysis of the differentially expressed metabolites

**Figure 10:**
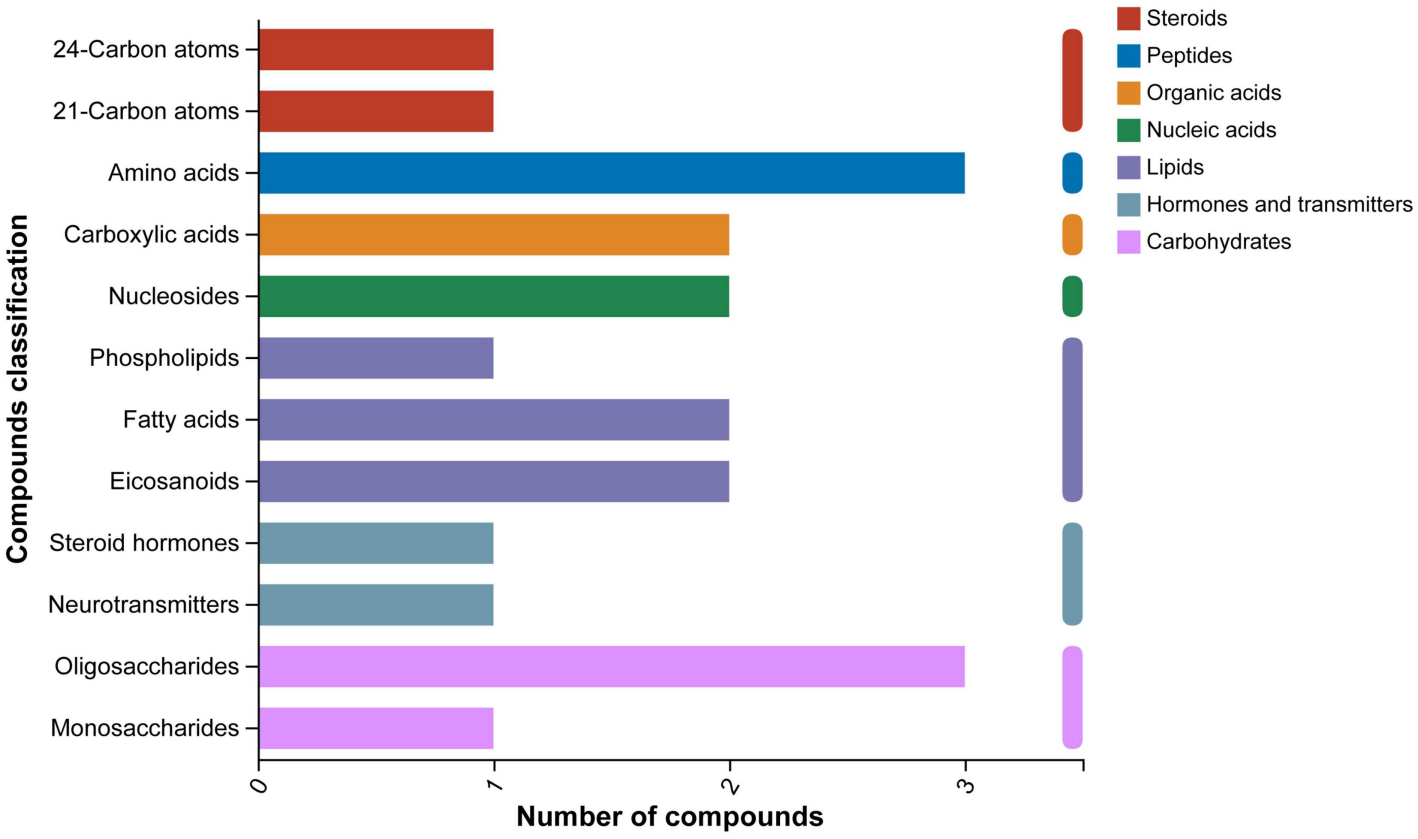
KEGG compound classification diagram of the differentially expressed metabolites between the OA + OP and OA + OP+LB-H groups.

**Figure 11:**
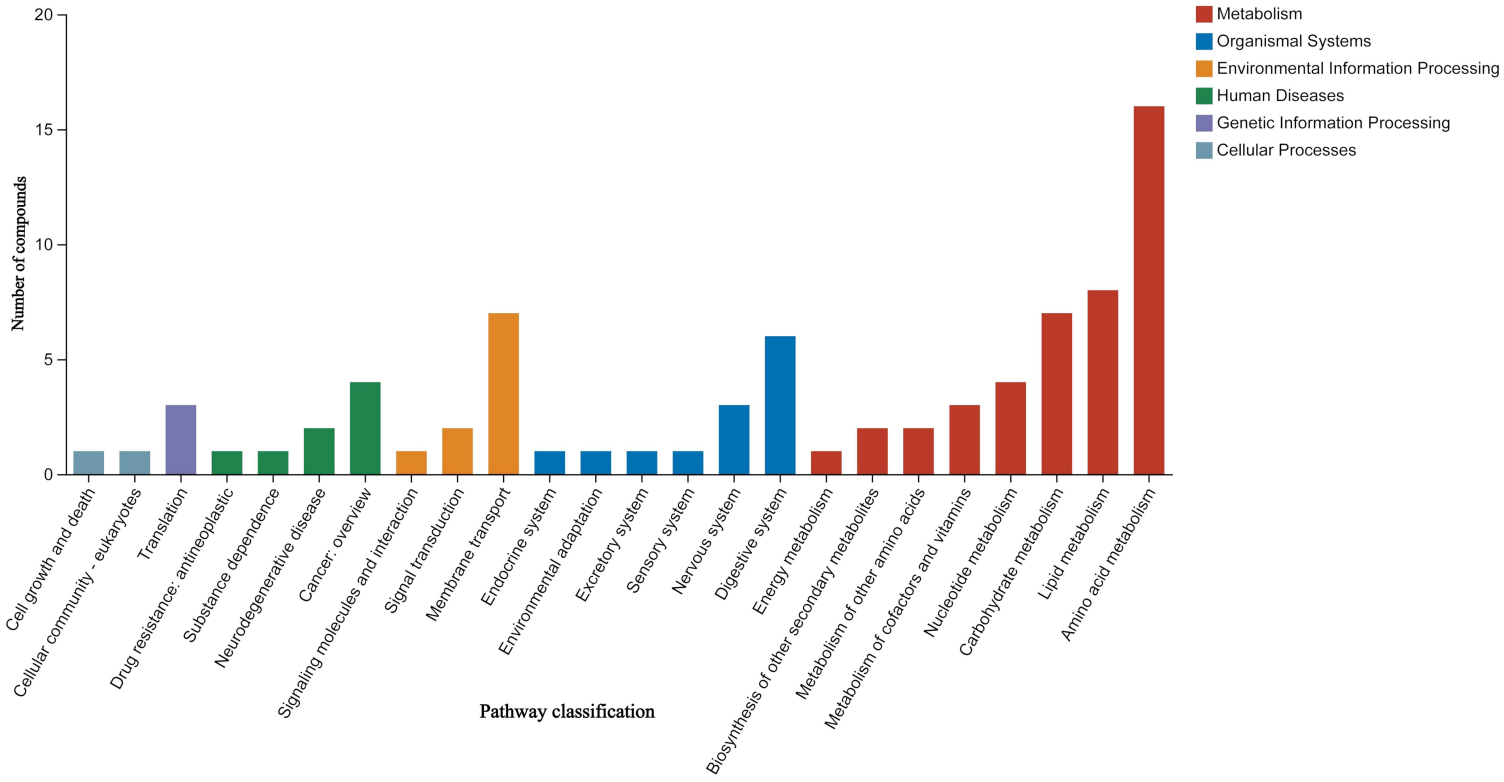
KEGG functional enrichment map of the differential expressed metabolites between the OA + OP and OA + OP+LB-H groups.

**Figure 12:**
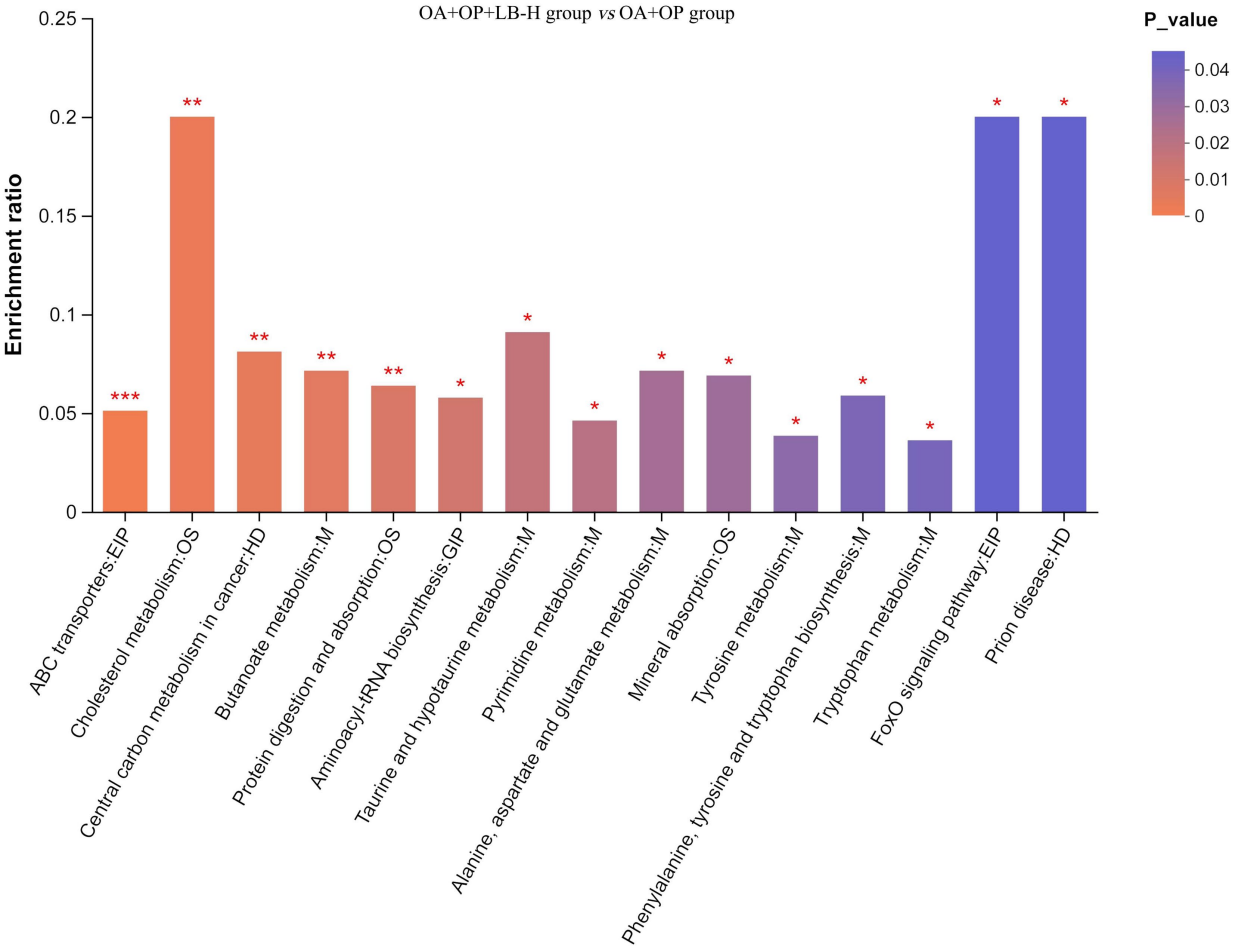
Enrichment map of the KEGG pathways for the differentially expressed metabolites between the OA + OP and OA + OP+LB-H groups. The color gradient of the column indicates the significance of enrichment. ***p-value or FDR < 0.001. **p-value or FDR < 0.01. *p-value or FDR < 0.05.

The classification results of the KEGG compounds for the differentially expressed metabolites are shown in Figure 10. The differentially expressed metabolites between the model group and the OA + OP+LB-H group were mainly classified as amino acids, oligosaccharides, carboxylic acids, and fatty acids. The results of the KEGG functional enrichment analysis indicate that the differentially expressed metabolites are mainly involved in amino acid metabolism, lipid metabolism, and carbohydrate metabolism (Figure 11). The KEGG pathway enrichment results are shown in Figure 12. The KEGG pathway enrichment results indicate that there are 34 entries for the differentially expressed metabolites involved in body metabolism (Supplementary material 3), such as butanoate metabolism; taurine and hypotaurine metabolism; pyrimidine metabolism; lysine degradation; and alanine, aspartate and glutamate metabolism. In terms of systemic organ systems, a total of 16 differentially expressed metabolites were involved (Supplementary material 3), such as in protein digestion and absorption, cholesterol metabolism, and mineral absorption. In terms of cellular processes, gap junction and ferroptosis were involved. The functional annotation results revealing environmental information processing indicate that the differentially expressed metabolites are mainly involved in pathways such as ABC transporters, the phospholipase D signaling pathway, the FoxO signaling pathway, and the cAMP signaling pathway.

## 4. Discussion

This study created a rat model of OA and OP comorbidity through bilateral oophorectomy combined with meniscus instability surgery and intervention with LBJN. We found that the main active ingredients of LBJN are tanshinone IIA, oleanolic acid, albiflorin, etc. The research results show that LBJN can improve the bone density and quality of subchondral bone in OA + OP rats, and LBJN can regulate the expression of BALP, OPG, and TRACP in serum, thereby affecting bone metabolism in OA + OP rats. Based on nontargeted metabolomics techniques and multivariate statistical analysis, 107 differentially expressed metabolites were found in the OA + OP+LB-H group compared to the OA + OP group. Among them, zeranol, 7,3′-dihydroxyflavone, 5-hydroxyindoleacetic acid, vanillactic acid, octanoylcarnitine, and TXB2 may be key targets through which LBJN exerts pharmacological effects. The enrichment analysis of KEGG pathways based on these differentially expressed metabolites suggests that the metabolic regulation pathway of LBJN treatment in OA + OP rats may be related to the cAMP signaling pathway and the FoxO signaling pathway.

OA and OP have multiple similar pathogenic factors, such as lipid metabolism disorders and high BMI (25). Under high BMI conditions, excessive release of adipokines and metabolic disorders can lead to an increase in OP and fracture risk. A higher BMI not only increases the mechanical load on the knee joint but also increases the risk of OA through adipose factor-induced aseptic inflammation (26). Therefore, abnormal lipid metabolism accompanied by pathological progression of OP may induce the occurrence and development of OA. This experimental study found that LBJN can regulate lipid metabolism and maintain the balance of bone metabolism. Inflammation and adipocytokines related to obesity may exacerbate the occurrence and progression of OP (27). Adipose tissue is considered an endocrine organ for bone tissue metabolism (28), but it can secrete various inflammatory cytokines and can have negative effects on bone tissue. LBJN may reduce bone loss in the subchondral bone of the knee by improving lipid metabolism disorders, which, to some extent, alleviates the progression of KOA. The regulation of amino acid metabolism is also a key pathway by which LBJN exerts therapeutic effects on OA + OP rats, among which the metabolite 5-hydroxyandoleacetic acid (5-HIAA) may be an important pharmacological target. 5-HIAA is a metabolite of 5-hydroxytryptamine (5-HT) that can represent the level of 5-HT. Research has shown that 5-HT is a key molecule in bone tissue dynamics (29, 30) and can regulate bone metabolism. In addition to the intestinal 5-HT contained in bone tissue, the osteoblasts, osteoclasts and osteocytes can also synthesize 5-HT (29). Yadav et al. showed that 5-HT can inhibit the proliferation of osteoblasts through the 5-HTR1B receptor on the surface of osteoblasts (31). Under normal physiological conditions, FoxO1 protein interacts with activating transcription factor 4 (ATF4) and cAMP responsive element binding protein (CREB) in the nucleus of osteoblasts to maintain normal proliferation. The binding of FoxO1 protein with ATF4 promotes the expression of transcription targets regulated by FoxO1, while binding with CREB inhibits the expression of transcription targets regulated by FoxO1. An increase in 5-HT levels in the blood circulation disrupts the interaction between FoxO1 and CREB (31, 32), which leads to a decrease in osteoblast proliferation activity. Therefore, we believe that LBJN may exert its anti-OP effect by regulating the cAMP/FoxO signaling pathway and acting on 5-HT.

Kidney-tonifying and blood-activating traditional Chinese medicine is a commonly used method for treating OA + OP (14, 33), and LBJN is a representative compound of this type of traditional Chinese medicine. LBJN has good clinical efficacy in the treatment of OA and has good efficacy in the treatment of OA in postmenopausal women. Postmenopausal women are at high risk of bone loss and OP due to a decrease in estrogen levels (34, 35). The imbalance in the activities of osteoblasts and osteoclasts may directly lead to the destruction of bone tissue structure and bone loss (36), and estrogen plays a key role in the balance of the activity of these two types of osteoblasts. The balance of bone metabolism in subchondral bone is also a major factor in maintaining cartilage stability (37). This study also found that LBJN can regulate estrogen metabolism and thereby play a role in improving cartilage quality, with the discovery of a key metabolite, zeranol. Zeranol is a new type of phytoestrogen that can bind to estrogen receptors and has estrogen and estrogen receptor antagonistic effects (38, 39). Estrogen can regulate various cytokines (such as IL-1 and IL-6) and can affect bone metabolism (40, 41). Research has shown that multiple subtypes of interleukin can be highly expressed in osteoporotic tissues, with IL-1 and IL-6 receiving significant attention (42, 43). IL-6 is a cytokine with extensive biological activity that is secreted by osteoclasts, osteoblasts, bone stromal cells and monocytes/macrophages (44) and plays a key role in the occurrence and progression of OP. IL-6 can stimulate the proliferation of osteoclasts and improve their functional expression (42), thereby promoting the occurrence of OP. IL-1 is an activator of osteoclasts and stimulates bone resorption (45). Osteoblasts can produce IL-6 and tumor necrosis factor (43, 45) under the stimulation of IL-1, and these three cytokines can jointly promote bone resorption. One study showed that a decrease in estrogen causes an increase in the IL-6 content (46) and then inhibits the apoptosis of osteoclasts. The FoxO/cAMP signaling pathway is also closely related to inflammation (47–49) and plays an important role in regulating the inflammatory microenvironment in obesity, OP, and OA. These findings indicate that the intervention effect of LBJN on OA + OP rats is characterized by impacting multiple pathways and targets.

This study has the following limitations. First, although we have identified the differentially expressed metabolites in the treatment of OA + OP with LBJN, the specific mechanism of action of LBJN has not yet been revealed. This will be the next step in this research and will be further explored in depth. Second, it is difficult to separate the components of LBJN and their related metabolites from metabolic samples containing a large amount of biological matrix, which may affect the accuracy of the conclusions of this study. Third, LBJN contains many potentially effective chemical components, but we cannot determine which components play the main pharmacological role, which represents an area for further exploration in future research. Finally, this study did not include an OP group, which did not allow us to compare the outcomes of LBJN treatment in an OP group versus the OA + OP group.

## 5. Conclusion

LBJN can maintain bone metabolism balance by regulating serum lipid metabolism, amino acid metabolism, carbohydrate metabolism and estrogen, further reducing bone loss in subchondral bone, which may be a potential mechanism of action for LBJN in treating OA + OP. The findings of this study provide evidence for the efficacy and mechanism of kidney-tonifying and blood-activating herbs in treating OA and OP comorbidities, but these findings depend on the future implementation of clinical randomized controlled trials for verification.

## Data availability statement

The raw data supporting the conclusions of this article will be made available by the authors, without undue reservation.

## Ethics statement

The animal study was approved by the Experimental Animal Center of Guangdong Provincial Academy of Chinese Medical Sciences. The study was conducted in accordance with the local legislation and institutional requirements.

## Author contributions

GL: Conceptualization, Formal analysis, Investigation, Writing – original draft, Writing – review & editing. JZ: Conceptualization, Formal analysis, Methodology, Writing – original draft, Writing – review & editing. DZ: Data curation, Methodology, Writing – original draft, Writing – review & editing. YD: Data curation, Formal analysis, Methodology, Software, Visualization, Writing – original draft, Writing – review & editing. HH: Formal analysis, Investigation, Software, Writing – original draft, Writing – review & editing. WY: Data curation, Formal analysis, Investigation, Writing – original draft, Writing – review & editing. GZ: Data curation, Investigation, Writing – original draft, Writing – review & editing. ZG: Data curation, Formal analysis, Software, Writing – original draft, Writing – review & editing. JP: Funding acquisition, Project administration, Supervision, Writing – original draft, Writing – review & editing. JL: Funding acquisition, Project administration, Supervision, Writing – original draft, Writing – review & editing.

## Footnotes

^1^ www.hmdb.ca

## Glossary

OA: Osteoarthritis
OP: Osteoporosis
LBJN: Longbie capsule
OA + OP: OA and OP comorbidities
BALP: Bone alkaline phosphatase
OPG: Osteoprotegerin
TRACP: Tartrate-resistant acid phosphatase
KOA: Knee osteoarthritis
BMD: Bone mineral density
HPLC-Q-Orbitrap-MS: High-performance liquid chromatography quadrupole/electrostatic field orbital trap high-resolution mass spectrometry
MS: Mass spectrum
ESI: Electric spray ionization
BV/TV: Bone volume fraction
Tb.N: Trabecular number
BS/BV: Bone surface area to bone volume ratio
Tb.Th: Trabecular thickness
Tb.Sp: Trabecular separation
ELISA: Enzyme-linked immunosorbent assay
UPLC–MS: Ultra-high-performance liquid chromatography-mass spectrometry
ISVF: Ion-spray voltage floating
DDA: Data-dependent acquisition
PCA: Principal component analysis
PLS-DA: Partial least squares discriminant analysis
OPLS-DA: Orthogonal partial least squares discriminant analysis
VIP: Variable importance in projection
HMDB: Human Metabolome Database
KEGG: Kyoto Encyclopedia of Genes and Genomes
